# Single-domain multiferroic BiFeO_3_ films

**DOI:** 10.1038/ncomms12712

**Published:** 2016-09-01

**Authors:** C.-Y. Kuo, Z. Hu, J. C. Yang, S.-C. Liao, Y. L. Huang, R. K. Vasudevan, M. B. Okatan, S. Jesse, S. V. Kalinin, L. Li, H. J. Liu, C.-H. Lai, T. W. Pi, S. Agrestini, K. Chen, P. Ohresser, A. Tanaka, L. H. Tjeng, Y. H. Chu

**Affiliations:** 1 Max Planck Institute for Chemical Physics of Solids, Nöthnitzer Str 40, 01187 Dresden, Germany; 2 Department of Materials Science and Engineering, National Tsing Hua University, Hsinchu 30013, Taiwan; 3 Department of Materials Science and Engineering, National Chiao Tung University, Hsinchu 30010, Taiwan; 4 Centre for Nanophase Materials Sciences, Oak Ridge National Laboratory, Oak Ridge, Tennessee 37831, US; 5 Multi-disciplinary Materials Research Center, Frontier Institute of Science and Technology, Xi'an Jiaotong University, Xi'an, Shaanxi 710049, China; 6 National Synchrotron Radiation Research Center, Hsinchu 30076, Taiwan; 7 Synchrotron SOLEIL, L'Orme des Merisiers, Saint-Aubin - BP 48, 91192 GIF-sur-YVETTE, France; 8 Department of Quantum Matter, ADSM, Hiroshima University, Higashi-Hiroshima 739-8530, Japan; 9 Department of Electrophysics, National Chiao Tung University, Hsinchu 30010, Taiwan; 10 Institute of Physics, Academia Sinica, Taipei 11529, Taiwan

## Abstract

The strong coupling between antiferromagnetism and ferroelectricity at room temperature found in BiFeO_3_ generates high expectations for the design and development of technological devices with novel functionalities. However, the multi-domain nature of the material tends to nullify the properties of interest and complicates the thorough understanding of the mechanisms that are responsible for those properties. Here we report the realization of a BiFeO_3_ material in thin film form with single-domain behaviour in both its magnetism and ferroelectricity: the entire film shows its antiferromagnetic axis aligned along the crystallographic *b* axis and its ferroelectric polarization along the *c* axis. With this we are able to reveal that the canted ferromagnetic moment due to the Dzyaloshinskii–Moriya interaction is parallel to the *a* axis. Furthermore, by fabricating a Co/BiFeO_3_ heterostructure, we demonstrate that the ferromagnetic moment of the Co film does couple directly to the canted moment of BiFeO_3_.

## Abstract (web summary)

The coupling of ferroelectric and antiferromagnetic order in BiFeO_3_ makes it appealing for applications however the presence of domain structure acts to undermine this potential. Here, the authors demonstrate BiFeO_3_ thin films with a single domain of electrical polarization and canted antiferromagnetic order.

## Graphical abstract

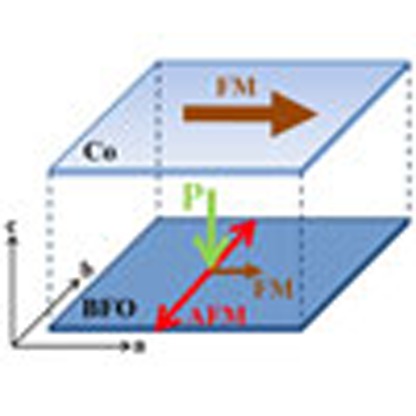

A promising approach to low-power, functional and green nanoelectronics relies on advances in the electric field control of lattice, charge, orbital and spin degrees of freedom in novel materials. Coexisting order parameters and inherent couplings in multiferroics form a potent playground, enabling the manipulation of the intriguing properties to obtain new functionalities using an electrical stimulus^1^. Among the numerous multiferroic systems, BiFeO_3_ (BFO) is the most promising and well studied^2^,^3^,^4^,^5^,^6^,^7^,^8^,^9^. BFO exhibits large ferroelectric polarization (**P**) and G-type antiferromagnetism with high transition temperatures, making it appealing for applications in nonvolatile logic and memory devices^10^,^11^. In spintronics, BFO has been used as block layer in spin valves^12^,^13^, with the aim of electrically controlling the giant magnetoresistance via the magnetoelectric coupling^12^. However, a long-range spin cycloid structure, as shown in Fig. 1a, met in bulk BFO limits its potential for practical applications^14^. Although this spin cycloid structure was reported to be suppressed in epitaxial BFO films^3^, there are still multi antiferromagntic (AFM) axes present as illustrated in Fig. 1b^3^,^4^,^15^. In fact, possible device applications are currently all demonstrated on samples with multi-domain structures^16^, which poses a limit for the device size. To significantly exploit the potential of BiFeO_3_ thin films, it is necessary to create films with single and well defined parameters for both the magnetic and ferroelectric orders as sketched in Fig. 1c.

The use of strain has emerged as a powerful tool for tailoring both the ferroelectric and the magnetic properties of BFO thin films^7^,^8^,^17^,^18^. While without strain a spin cycloidal modulation was observed^8^, the presence of compressive strain rotates **P** towards the *c* axis and the AFM axis towards the *ab*-plane^8^,^18^,^19^,^20^,^21^,^22^. In fact, the AFM axis is inside the *ab*-plane for *c/a* > 1.04 (ref. 8). In this work, we explore the use of NdGaO_3_ (NGO) as substrate which gives the BFO film not only a large in-plane compressive strain of *c/a*∼1.08 but also an in-plane anisotropic strain with non-identical *a* axis (∼3.86Å) and *b* axis (∼3.87 Å) as to find the route to prepare a single ferroelectric and antiferromagnetic domain BFO film. Utilizing a thin LaNiO_3_ (LNO) bottom electrode buffer and staying below a certain film thickness, we indeed achieve the single multiferroic domain BFO film, which has a single AFM axis parallel to *b* axis and a single **P** nearly towards *c* axis as well as a canted ferromagnetic moment due to the Dzyaloshinskii–Moriya interaction along *a* axis for entire BFO film. The realization of the single-domain multiferroic BiFeO_3_ thin films provides insights into the fundamental interactions of BiFeO_3_ and opens a promising path for engineering novel functional devices.

## Results

### Study of AFM axis

**Figure 1:**
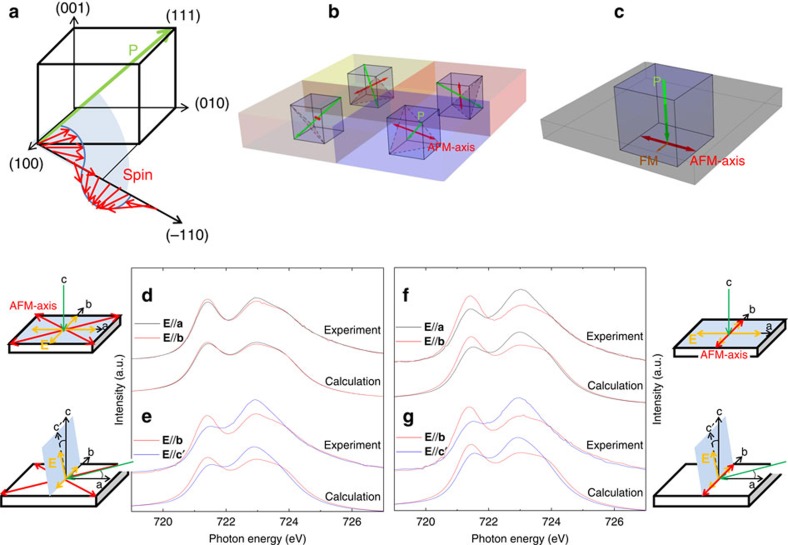
AFM structure of BFO bulk and thin films. (**a**–**c**) Schematic of spin structure of (**a**) bulk BFO, (**b**) a multi AFM axes BFO film and (**c**) a single AFM axis BFO film. (**d**–**f**) Experimental and calculated polarization-dependent Fe-L_2_ XAS spectra of a 200Å BFO film (**d**,**e**) and a 40 Å BFO film (**f**,**g**). The experimental geometries are depicted next to each set of spectra. The Poynting vector (green arrow) and the **E** vector (yellow arrow) of the incoming light, as well as AFM axes (red arrow) are also indicated.

The magnetic properties of BFO film were studied by polarization-dependent soft X-ray absorption spectroscopy (XAS) at the Fe-L_2,3_ edges. A 10 Å LaNiO_3_ (LNO) bottom electrode is deposited between BFO film and NGO substrate. The bottom panel of Fig. 1 shows the Fe-L_2_ XAS of two BFO/LNO/NGO thin films. Figure 1d,f shows the spectra measured with **E**//**a** (black line) and **E**//**b** (red line) with the incident beam along the *c* axis. Figure 1e,g shows the spectra measured with **E**//**b** (red line) and **E**//**c′** (blue line) with the incident beam 20^o^ grazing from the *a* axis. The line shape of the spectra depends strongly on the relative orientation of the electric field vector of the light, the crystallographic axes and the spin orientation of the magnetic ions^23^,^24^,^25^. For the experimental geometries used here and the present case of Fe ions having the high-spin half-filled shell configuration, the polarizations dependence contains direct information about the spin orientation^23^,^24^,^25^. We define the X-ray magnetic linear dichroism (XMLD) spectrum here as the difference of the spectra taken with two orthogonal polarizations.

For the 200 Å BFO film, we observe a strong polarization dependence between **E**//**b** and **E**//**c′** as shown in Fig. 1e, while the difference between **E**//**a** and **E**//**b** is negligible as shown in Fig. 1d. With the AFM axis lying in-plane, this observation indicates the presence of a multi AFM axes state, since the spot size of the incident light in our experiments (∼1 mm^2^) is much larger than the AFM domains (∼50 nm^2^) of BFO films^3^. For the 40 Å BFO film, we see a very large difference between the **E**//**a** and **E**//**b** spectra as shown in Fig. 1f. Furthermore, the size of this in-plane XMLD signal is almost same as the out-of-plane XMLD signal measured between **E**//**b** and **E**//**c′** shown in Fig. 1g. This strongly suggests that we have succeeded in stabilizing a single-domain AFM system with the axis oriented along the *b* axis.

To extract accurate information about the AFM axis from the polarization-dependent Fe-L_2_ XAS, we have carried out simulations using the well established configuration interaction cluster calculations. For the 200 Å BFO film, we considered two nearly orthogonal AFM axes lying in the ab-plane. For the 40 Å BFO film, we took a single AFM axis parallel to *b* axis. One can see from Fig. 1d–g that all experimental spectra can be nicely well reproduced by our calculations confirming the change from multi AFM axes in the 200 Å BFO film to a single AFM axis state in the 40 Å BFO film. Further simulations (see Supplementary Fig. 1 and Supplementary Note 1) show that if the spin is considered along the *a* axis in the 40 Å BFO film, we can get the XMLD signal opposite to the experiment. To further confirm our findings, we have also measured the angular dependence of the XMLD (see details in Supplementary Note 2). We observe that the largest XMLD signal is found between **E**//**a** and **E**//**b** by rotating the sample around the c axis, or between **E**//**b** and **E**//**c′** by rotating sample around the *a'* axis (Supplementary Fig. 2). All these XMLD spectra can be also well reproduced by our simulations with the AFM axis fixed around the *b* axis. Furthermore, if we rotate the sample along the *b'* axis (Supplementary Fig. 3), both the experimental and theoretical results show nearly zero XMLD signal. The similar XAS for **E**//**a** and **E**//**c′** (see also in Supplementary Fig. 3) could be interpreted in term of an AFM axis along [101]. However, we can firmly exclude this possibility (see Supplementary Figs 4 and 5 and Supplementary Note 3). All of these results reveal that the spin direction of this single AFM axis state in the 40 Å BFO film is parallel to the *b* axis. Since the large XMLD signal between **E**//**a** and **E**//**b** has the AFM origin, it can be used to determined the Neel temperature, which is found to be around 450K for this single AFM axis state BFO film (Supplementary Fig. 6).

### Characterization of ferroelectric properties

**Figure 2:**
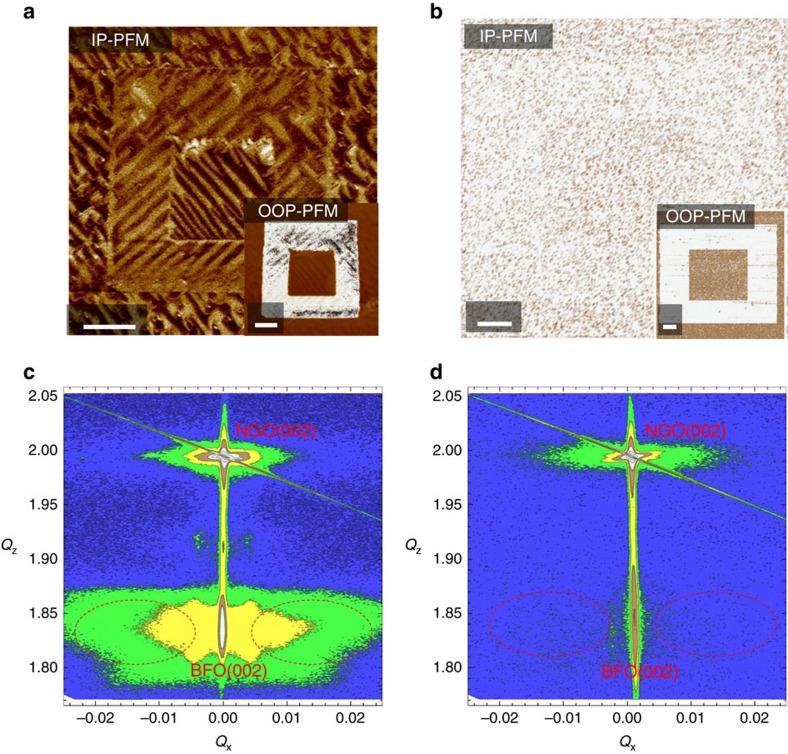
Ferroelectricity of multi-domain and single-domain BFO films. (**a**,**b**) In-plane (IP) and out-of-plane (OOP) PFM images of 200 Å (**a**) and 40 Å (**b**) BFO films. Scale bars, 1.0 μm (**c**,**d**) RSM results of the 200 Å (**c**) and 40 Å (**d**) BFO films around the (002) Bragg peak.

Having established the single-domain AFM structure, we now probe its ferroelectric properties by utilizing the piezo response force microscopy (PFM). In the upper panel of Fig. 2, the domains with up- and down-polarizations give rise to opposite contrast in the out-of-plane (OOP)-PFM images, and the differences in the in-plane components of **P** produce different torques on the cantilever of the atomic force microscope leading to the contrast in the in-plane (IP)-PFM images. By combining the OOP- and IP-PFM images, we can determine the **P** direction. The three contrast levels observed in IP-PFM image of 200 Å BFO film as shown in the Fig. 2a indicate that its domain structure is characterized by four ferroelectric **P** variants. The uniform contrast in the OOP-PFM image shown in the inset figure of Fig. 2a indicates that all these four **P** share the same OOP component that is downward to the LNO bottom electrode. For the 40 Å BFO film on the other hand, both the uniform OOP-PFM and IP-PFM images as shown in Fig. 2b indicate a single downward ferroelectric **P** domain. The nearly zero lateral contrast of the atomic force microscope cantilever found for the in-plane components means that the **P** direction is nearly parallel to the *c* axis.

X-ray reciprocal space mapping (RSM) is also used to characterize the BFO thin films. Fig. 2c shows the RSM of the 200 Å BFO film around the (002) Bragg peak. The large splitting between the main peak of the BFO film and the NGO substrate indicates the large in-plane compressive strain with a *c/a* ratio of ∼1.08. One can clearly see that there is a wing structure around the (002) peak. This wing arises from the periodic domain structure, thus reflecting the multi ferroelectric **P** domain state^7^,^26^. For the 40 Å BFO film by contrast, the wing structure around the (002) peak is completely absent as shown in Fig. 2d, indicating the single ferroelectric **P** domain. This is fully consistent with the above mentioned PFM and XMLD results.

## Discussion

What is the mechanism for driving **P** parallel to the *c* axis and the AFM axis along the *b* axis in the 40 Å BFO thin film grown on NGO substrate? We have found four ingredients to achieve the single-domain state: (1) in-plane compressive strain, (2) in-plane lattice anisotropy, (3) bottom electrode and (4) limited film thickness.

**Figure 3:**
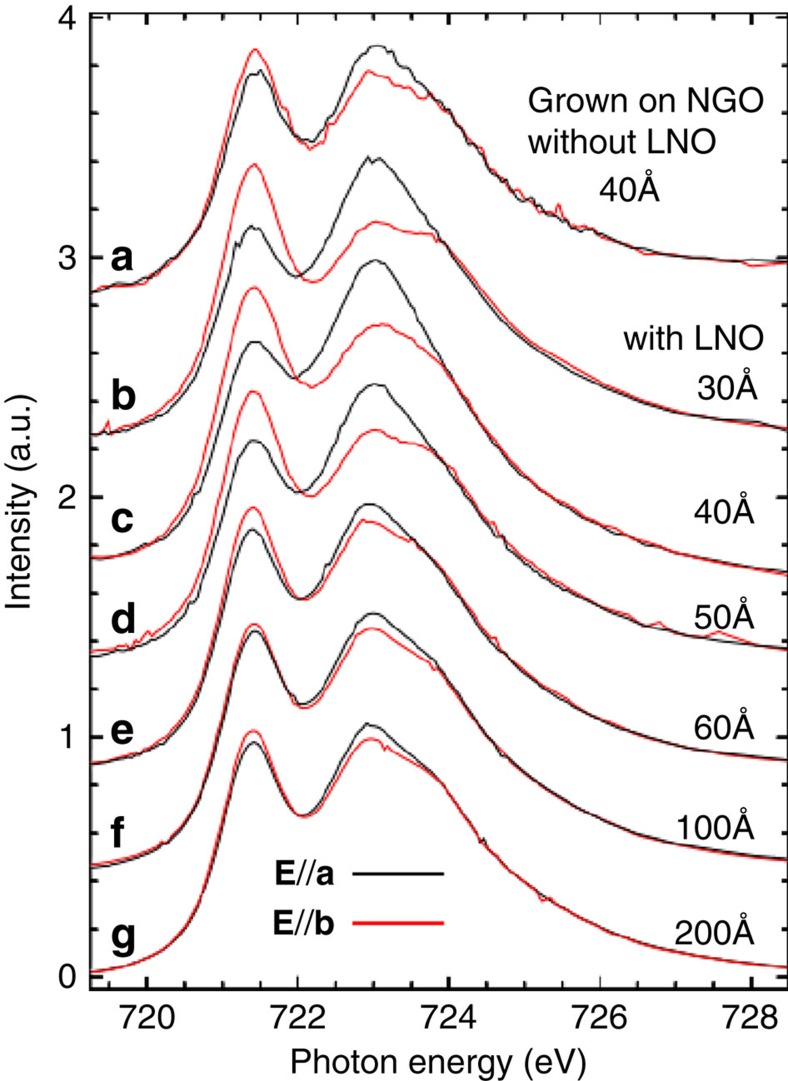
Experimental Fe-L_2_ XAS spectra of different BFO films. (**a**) A 40 Å BFO film grown on NGO without LNO bottom electrode. (**b**–**g**) Different thickness (30–40–50–60–100–200 Å) BFO films grown on NGO with LNO bottom electrode.

Previous experimental and theoretical studies demonstrated that for an in-plane compressive strain, the **P** will rotate away from the [111] towards the [001] direction^18^,^19^,^20^. When the *c/a* ratio exceeds 1.1, the **P** has been found to be almost parallel to the *c* axis^21^,^22^. A BFO film grown on the NGO substrate with *c/a* of ∼ 1.08 has **P** close, but not parallel to *c* axis. This has consequences for the AFM domains. Figure 3a shows the XAS spectrum for a 40 Å BFO grown on the NGO but without the LNO bottom electrode. The difference between the **E//a** and **E//b** spectra is rather small, indicating a multi AFM axes structure, which is in turn in accordance with the multi **P** domain structure as revealed by PFM (see Supplementary Figure 7 and Supplementary Note 4).

Apparently we need **P** to be more parallel to the *c* axis in order for the in-plane magneto-crystalline anisotropy to have sufficient effect. The alignment of **P** can be done by inserting a bottom electrode layer of LNO. The free charges in bottom electrode, which have the opposite sign to the surface charges of ferroelectric, would like to approach ferroelectric-electrode interface^27^,^28^,^29^ leading to a ‘built-in electric field'^30^. To confirm such effect of LNO, we measured the vertical PFM phase loop as the function of DC voltage for BFO(40 Å)/LNO/NGO as shown in Supplementary Fig. 8. A clearly observable voltage bias suggests a presence of downward electric field^29^, that is believed to further align **P** to *c* axis. Important then is to be aware that the effectiveness of the LNO may be a function of the thickness of the film. Figure 3b–g shows the Fe-L_2_ XAS spectra with thicknesses ranging from 30 to 200 Å. We observe clearly that the difference between the **E//a** and **E//b** spectra diminishes with increasing thickness indicating that the single-domain state cannot survive if the film is thicker than about 50 Å. We attribute this diminishing effect with thickness to the depolarization energy, which can be approximately proportional to *σ*^*2*^*t* for keeping single **P** along *c* axis^31^. Here *σ* is the surface charge density induced by **P** 28, and *t* is the thickness of the BFO film. The depolarization energy for **P** parallel to *c* axis then increases with BFO film thickness and competes with the effect given by LNO. For the thick BFO film, it is thus favour to form the multi-domain structure, which has **P** deviated from *c* axis, to reduce the surface charge density *σ* and consequently save the depolarization energy.

It is interesting to analyse the origin of the in-plane magneto-crystallinity. One could expect that it is negligibly small, since the Fe^3+^ of BFO has the ^6^S or ^6^A_1_ ground state with a completely quenched orbital momentum. However, a small amount of orbital momentum can be induced by mixing higher lying states via the spin-orbital coupling and the Fe 3*d*–O 2*p* hybridization^32^,^33^. A small magnetic anisotropy can thus be induced by the presence of an anisotropic crystal field, which in turn can stabilize a uniaxial AFM axis. The classical example for this is the α-Fe_2_O_3_ system where a rotation of the AFM axis by 90° was observed at a relatively high temperature of 263 K, known as the Morin transition^23^. It was estimated that the magnetic anisotropic energy in α-Fe_2_O_3_ is in the range of several μeV (ref. 34). A similar value can be found for BFO^35^. In other words, the in-plane crystal anisotropy induced by the NGO substrate can indeed be used to generate the single AFM axis state.

**Figure 4:**
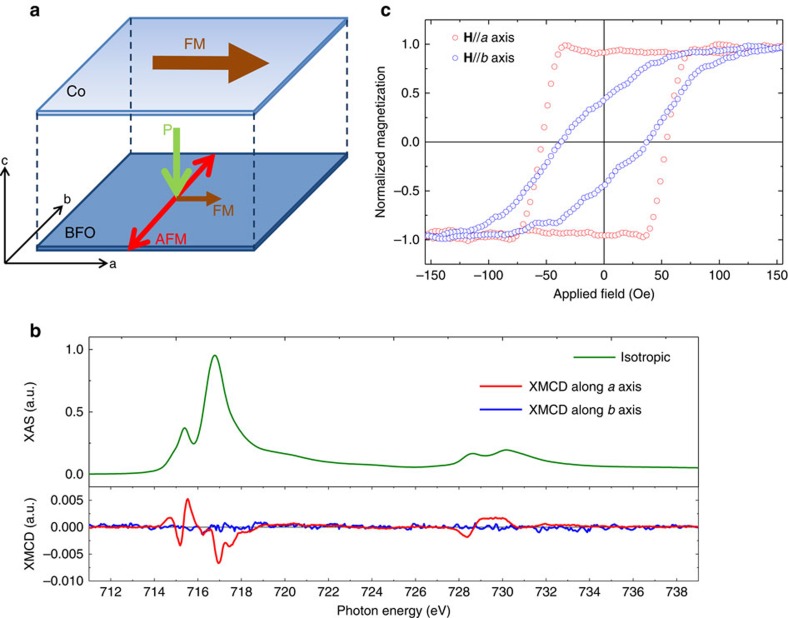
Ferromagnetism properties of single-domain BFO film. (**a**) Schematic of magnetization and ferroelectric polarization axes for a heterostructure consisting of a 30 Å Co metal film grown on a single-domain 40 Å BFO film. (**b**) Isotropic Fe-L_2,3_ XAS (green line) and XMCD spectra of BFO(40 Å)/LNO/NGO taken with the incident light along the *a* axis (red line) and *b* axis (blue line), and remnant magnetic field. (**c**) Magnetic hysteresis loop of the Co(30 Å)/BFO(40 Å) heterostructure with magnetic field along the *a* axis (red circles) and *b* axis (blue circles).

Various theoretical studies have proposed that a weak FM moment can be induced in BFO films along the direction perpendicular to both **P** and the AFM axis via the Dzyaloshinskii–Moriya (DM) interaction^36^,^37^,^38^. With a single-domain BFO film at hand, we can now test reliably this presence of ferromagnetism. With the AFM axis parallel to the *b* axis and **P** parallel to the *c* axis, we expect the FM moment to be along the *a* axis as illustrated in Fig. 4a. We have carried out XAS and X-ray magnetic circular dichroism (XMCD) measurements at the Fe-L_2,3_ edges. Figure 4b shows the isotropic spectrum (green line). The XMCD spectrum, which is defined as the difference between spectra taken with the photon helicity parallel and antiparallel to the remnant magnetic field in the film, is also shown in Fig. 4b. The red line is for the spectrum where the Poynting vector of the light makes an angle of 20° with the *a* axis. We can clearly observe an XMCD signal. Measuring with the Poynting vector at an angle of 20° with the *b* axis, on the other hand, resulted in a vanishing XMCD signal, see the blue line. These results thus firmly establish that the FM moment induced by the DM interaction in this single AFM axis BFO film is along the *a* axis.

To lead to potential applications, it is important to demonstrate that an exchange coupling between the single-domain BFO film and a FM material. To this end we have created a heterostructure consisting of a 30 Å polycrystalline Co film deposited on top of the 40 Å BFO film. Fig. 4c shows the magnetic hysteretic loop of this Co/BFO bilayer measured by a vibrating sample magnetometer. A very clear magnetic anisotropy can be observed with the easy axis aligned along the *a* axis, that is, perpendicular (parallel) to the AFM axis (FM moment) of the BFO film. This provides direct evidence that we indeed can make use of the unique properties of the single-domain BFO film to steer the magnetic orientation of a FM material.

In summary, we have demonstrated a route to make single-domain multiferroic BFO films. Using the large in-plane compressive strain with crystal anisotropy, built-in electric field from a bottom electrode, and an appropriate film thickness we were able to force the electrical polarization **P** to be oriented nearly along the *c* axis and the AFM axis along the *b* axis. We have also successfully identified and coupled the resulting FM moment along the *a* axis via an exchange coupling to a FM material, thereby demonstrating the potential of this heterostructure for applications. The next step is to realize the electrical control of the FM moment in the entire thin film (∼25 mm^2^), and then show the occurrence of magnetoelectricity using bulk magnetic measurements. For the large-area (∼25 mm^2^) sample, however, our present electrical switching experiments suffer from leakage problems, which preclude the **E**-field building up on the BFO film. For this we have to improve further the quality of the ultrathin film growth process in order to minimize the presence of particulates and defects as to avoid electrical leakage.

## Methods

### Sample preparation

Epitaxial BFO and LNO thin films were fabricated on NGO(001)_pc_ single crystal substrate via pulsed laser deposition, with a KrF (*λ*=248 nm) excimer laser. The pulsed laser beam was focused on BiFeO_x_ ceramic target with an energy density of ∼ 2.5 J cm^−2^ and repetition rate of 10 Hz. Samples were deposited at the substrate temperature of 700 °C and with 100 mTorr oxygen pressure. Samples were cooled to room temperature in an oxygen pressure of 1 atm after thin film deposition.

In the whole manuscript, we use the pseudo-cubic system in which the [100]_pc_, [010]_pc_ and [001]_pc_ (blue arrows in Supplementary Fig. 9(a)) are referred to [001]_o_, [1-10]_o_, and [110]_o_ in the orthorhombic system (grey arrows in Supplementary Fig. 9(a)), respectively. Our BFO is grown on the NGO(001)_pc_. As the RSM results of BFO/LNO/NGO shown in the Supplementary Fig. 9(b), the BFO film grown on LNO-buffered NGO is in a nearly fully strain state. So in the presence of the LNO bottom electrode, NGO can play its role to induce the in-plane lattice anisotropy of BFO film with *a* axis ∼3.86 Å and *b* axis ∼3.87 Å.

### Absorption spectroscopy

The XMLD spectra were carried out at the 08B beamline of the National Synchrotron Radiation Research Center (NSRRC) in Taiwan. A single crystal Fe_2_O_3_ was measured simultaneously for energy calibration. XMCD measurements were carried out at the DEIMOS beamline of SOLEIL in Paris^39^.

### Configuration interaction cluster calculations

This theoretical approach includes the full atomic multiplet theory and the local effects of the solid^40^,^41^. It takes into account the intra-atomic and local crystal field interactions as well as the Fe 3*d*–O 2*p* hybridization^42^. The parameters [eV] used in FeO_6_ cluster calculations for BFO film are: 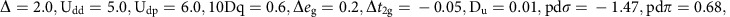
and the Slater integrals reduced to 70% of the Hartree Fock values.

### PFM analyses

Band excitation (BE) PFM was performed at the CNMS using an Asylum Research Cypher (commercial AFM), custom equipped with PXi data acquisition cards from National Instruments to enable band excitation measurements. The BE measurements were conducted via in-house scripts written in Labview and Matlab. All analysis of PFM data was carried out in Matlab v2013b. Measurements were conducted at room temperature in ambient humidity with Budget sensors Cr/Pt coated ElectriMulti75-G tips with nominal force constant of 3 N m^−1^.

The in-plane PFM response is weak for 40 Å BFO. Therefore, we have used the BE PFM to enhance the sensitivity of the in-plane component (by the Q-factor, usually around the 80–150). Supplementary Fig. 10 shows our in-plane PFM image measurement of 40 Å BFO grown on DyScO_3_ and SrTiO_3_ substrate with bottom electrode SrRuO_3_ as examples. One can see clearly the multi-domain structure. There are also reports on detectable in-plane PFM response of ultrathin BFO films^43^,^44^. Therefore, to detect the ferroelectric signal for such a thickness is not difficult. In our BFO(40 Å)/LNO/NGO data, we saw neither the in-plane PFM contrast nor the lateral resonance. Instead, we observed the clearly out-of-plane PFM response. We then conclude that the 40 Å BFO thin film grown on NGO shows single domain with the polarization nearly close to out-of-plane.

### Magnetic hysteresis loops

The ferromagnetic Co (3 nm) layer is deposited by dc magnetron sputtering at room temperature. A layer of Ta (∼8 nm) is capped on Co layer. The base pressure of sputtering is 7 × 10^−8^ Torr. Working pressure and plasma energy density of Co is 0.5 mTorr and 2.8 W in^2^. The hysteresis loops are measured by a vibrating sample magnetometer (Micromag 3900) at room temperature. To verify the presence of the magnetic coupling between the Co and the BFO films, we measured the magnetic anisotropy of a Co film grown on LNO/NGO without and with BFO film as shown in the Supplementary Fig. 11(a,b), respectively, where both samples were fabricated at the same time. One can see that Co grown without BFO shows the isotropic magnetic hysteresis loop. Under the same growth condition for Co film grown on a 200 Å BFO film which has the multi-domain state, Co exhibits also isotropic magnetic hysteresis loop as shown in Supplementary Fig. 11(c). This demonstrates that to have the magnetic anisotropy in the Co metal film, the Co has to be coupled with the net FM in a single-domain BFO thin film.

### Data availability

The data that support the findings of this study are available from the corresponding authors on request.

## Additional information

**How to cite this article:** Kuo, C.-Y. *et al*. Single-domain multiferroic BiFeO_3_ films. *Nat. Commun.* 7:12712 doi: 10.1038/ncomms12712 (2016).

## Supplementary Material

Supplementary Information Supplementary Figures 1-11, Supplementary Notes 1-4 and Supplementary References
