# Supplementary material for: Single-domain multiferroic BiFeO3 films
Source: Nat Commun. 2016 Sep 1;7:12712. doi: 10.1038/ncomms12712 (PMC5025802; doi:10.1038/ncomms12712)
Supplement: Supplementary Information — Supplementary Figures 1-11, Supplementary Notes 1-4 and Supplementary References [file ncomms12712-s1.pdf]

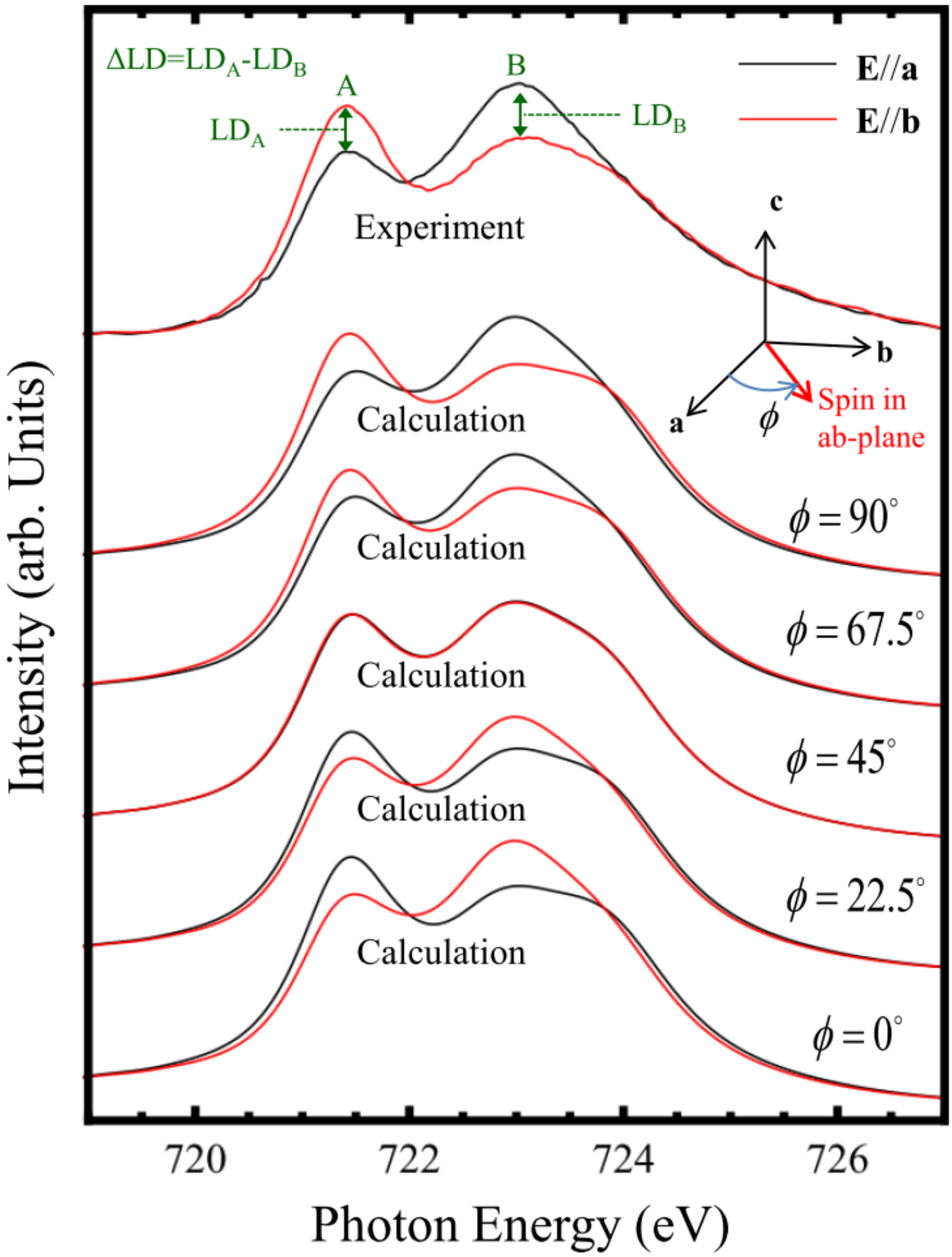

Supplementary Figure 1| Spin orientation dependent X-ray absorption spectroscopy (XAS). Experimental Fe-L_2 XAS spectra of the BFO(40Å)/LNO/NGO film for **E**//**a** (black line) and for **E**//**b** (red line), together with calculations in which the direction of the spin is varied from $\phi = 90°$ (parallel to the *b*-axis) to $\phi = 0°$ (parallel to the *a*-axis).

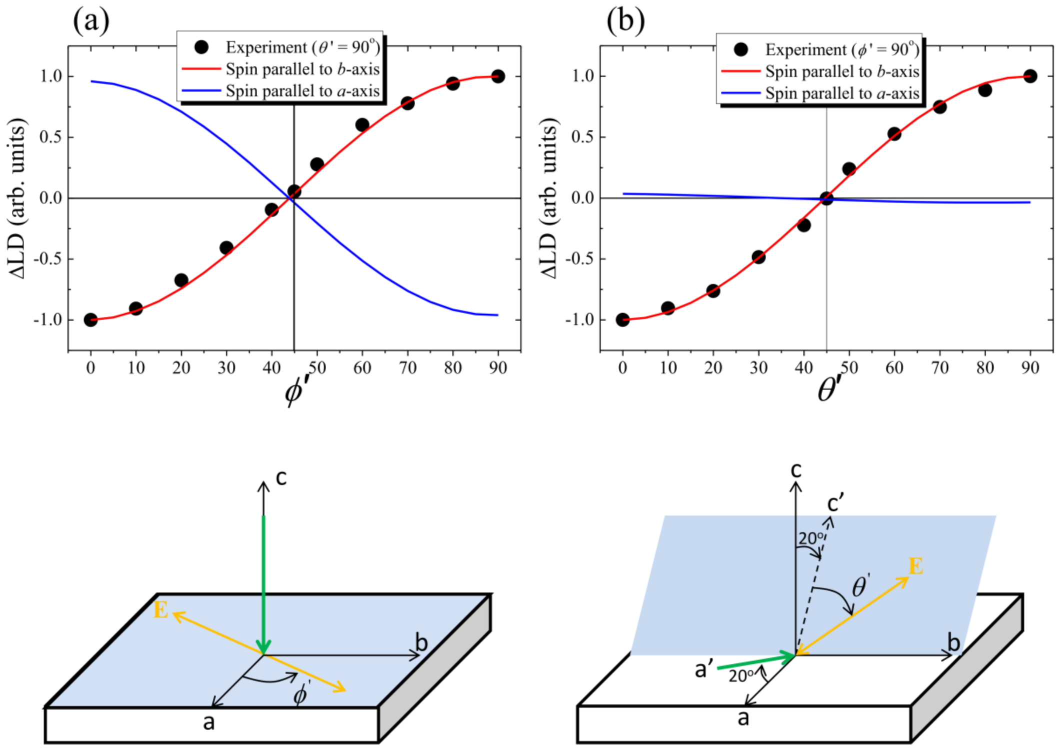

Supplementary Figure 2| Angle dependent ΔLD of BFO(40Å)/LNO/NGO. The experimental (black circles) and calculated polarization dependence ΔLD as function of ϕ' (a) and of θ' (b) for spin parallel to *b*-axis (red line) and spin parallel to *a*-axis (blue line). The measurement geometries are depicted at the bottom of the figure. The Poynting vector (green arrow) and the **E** vector (yellow arrow) of the incoming light are also indicated.

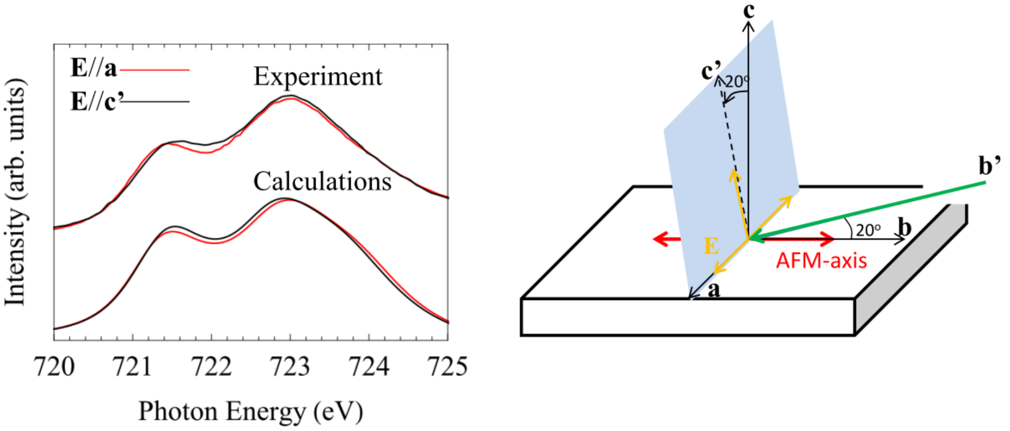

Supplementary Figure 3| Linear polarized X-ray absorption spectroscopy (XAS) of BFO(40Å)/LNO/NGO. The experimental and calculated Fe-L_2 XAS spectra for **E**//**a** (red) and **E**//**c**' (black) with the Poynting vector (green arrow) at a 20° grazing angle with the *b*-axis. The measurement geometry is depicted at the right side of figure. The Poynting vector (green arrow) and the **E** vector (yellow arrow) of the incoming light are also indicated.

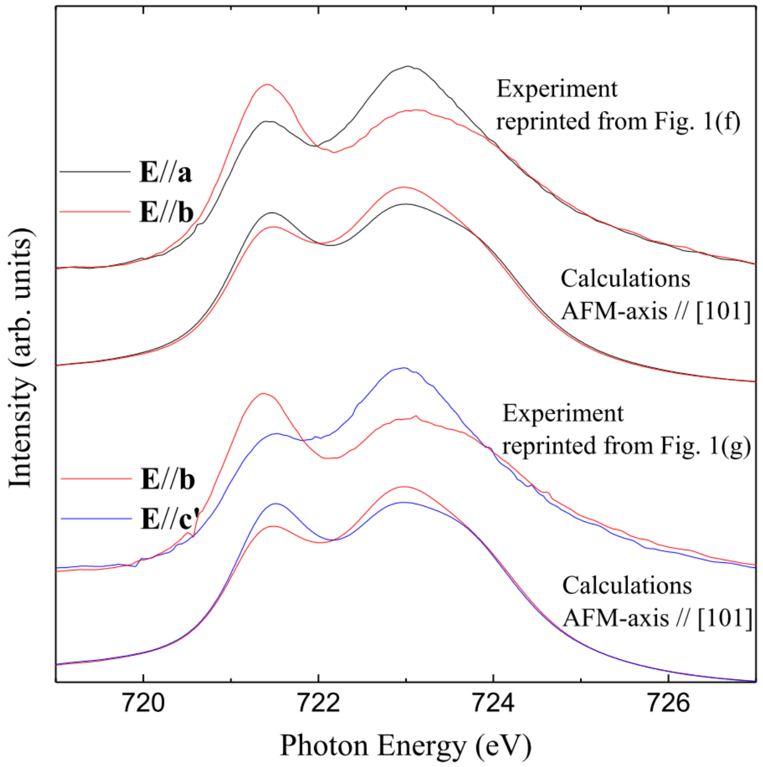

Supplementary Figure 4| Linear polarized X-ray absorption spectroscopy (XAS) of BFO(40Å)/LNO/NGO. The experimental spectra are reprinted from Fig.1(f,g) of the main text. The calculations are done with AFM-axis parallel to [101].

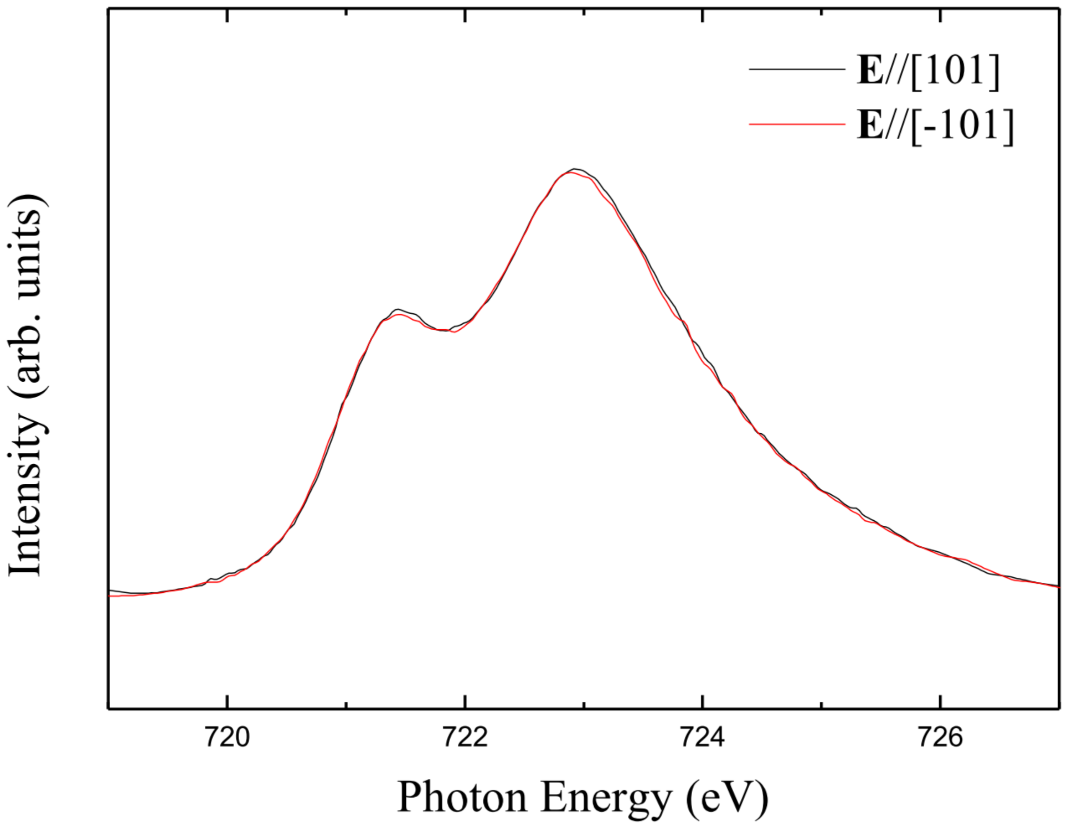

Supplementary Figure 5| Linear polarized X-ray absorption spectroscopy (XAS) of BFO(40Å)/LNO/NGO. Spectra were measured for **E**//[101] (black line) and **E**//[-101] (red line).

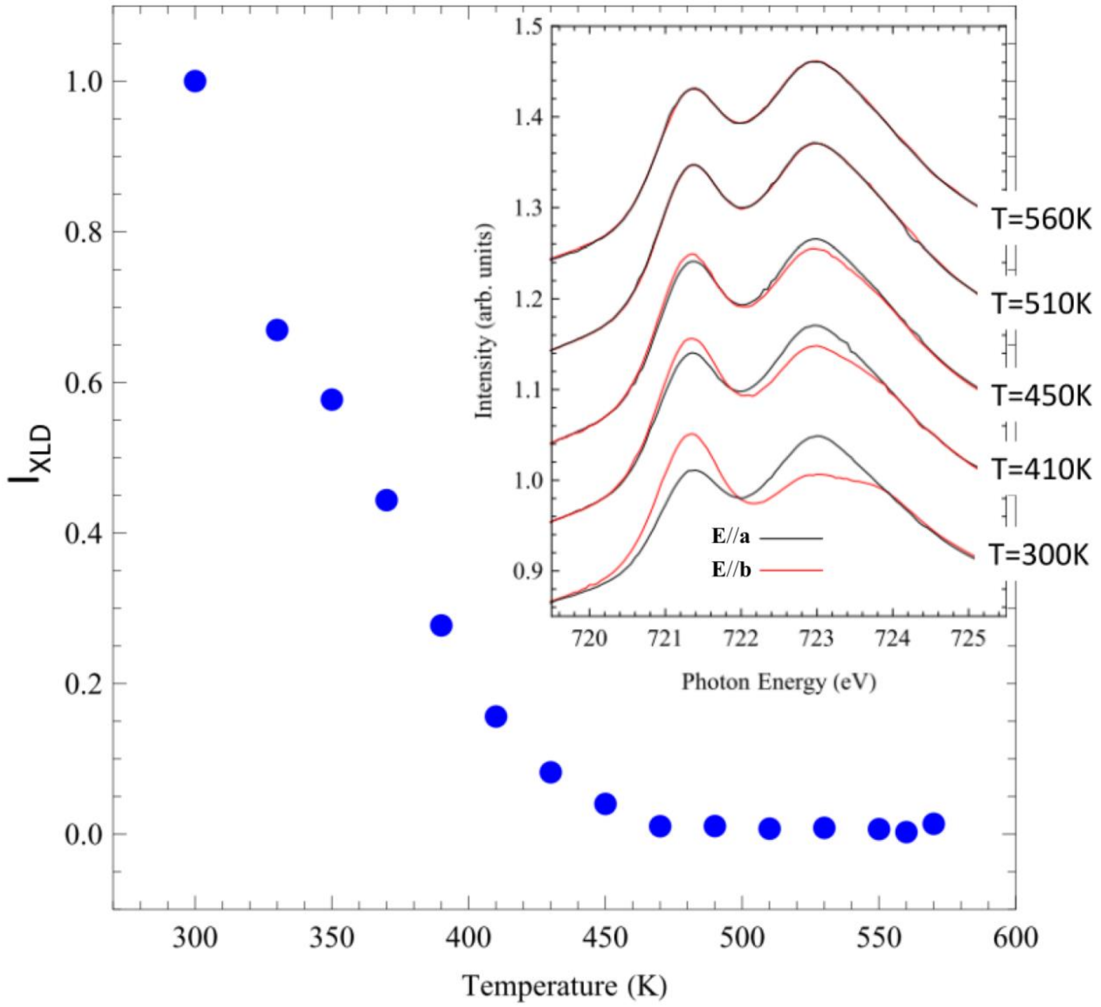

Supplementary Figure 6| Temperature dependent I_{XLD} of BFO(40Å)/LNO/NGO. I_{XLD} is the integration of the absolute XLD signal |**E**//**a**-**E**//**b**|^2 from 720 eV to 725 eV (see the inset). The inset shows polarization dependent XAS spectra between **E**//**a** (black line) and **E**//**b** (red line) at different temperatures.

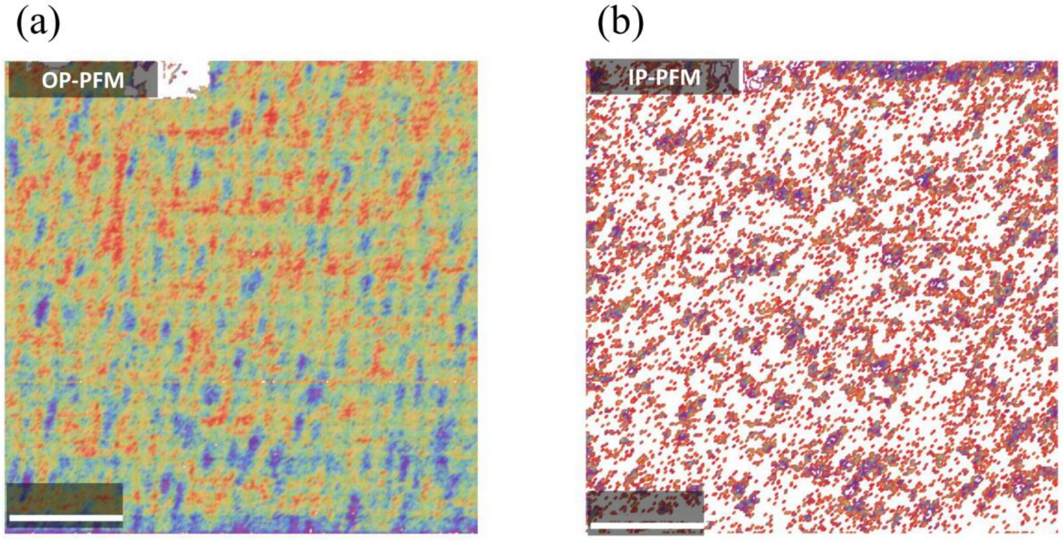

Supplementary Figure 7| Piezo response force microscopy (PFM) images of BFO(40Å)/NGO. (**a**) Vertical (OP-) PFM image, (**b**) Lateral (IP-) PFM image. Scale bars, 0.5um.

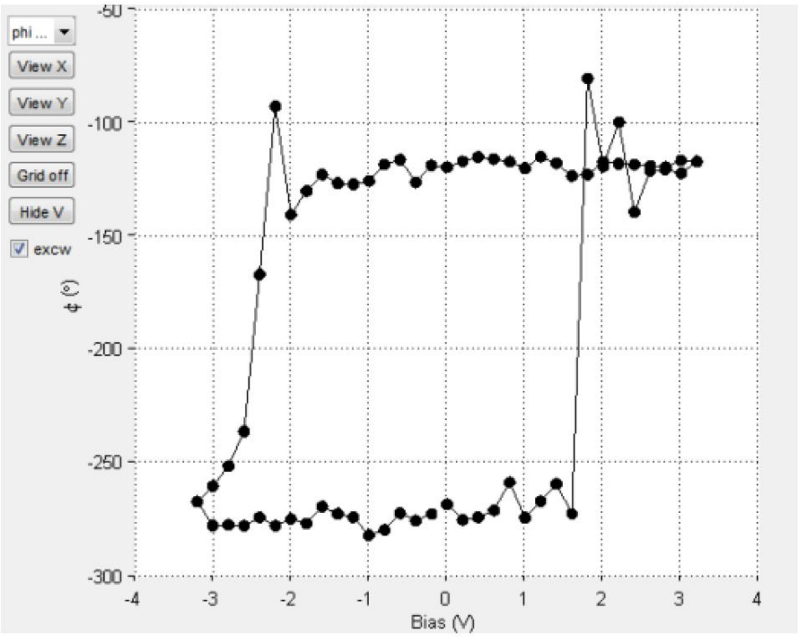

Supplementary Figure 8| The vertical PFM phase loop as the function of DC voltage for BFO(40Å)/LNO/NGO. A clearly observable voltage bias suggests a presence of downward electric field induced by bottom electrode and called "built-in electric filed" in our work.

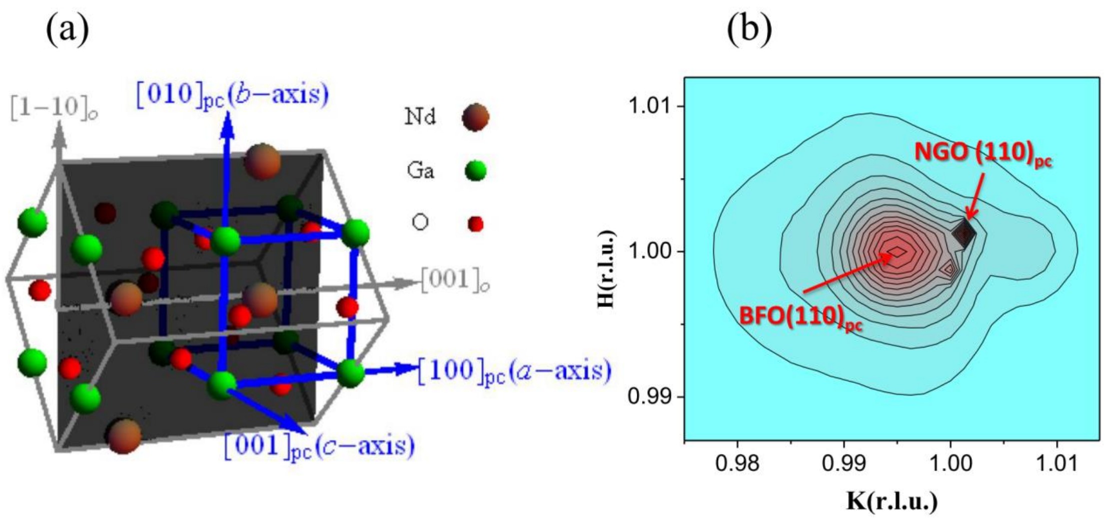

Supplementary Figure 9| Pseudo-cubic definition of $NdGaO_3$ (NGO) and $BiFeO_3$ (BFO). (**a**) Illustration of the definitions with respect to orthorhombic system (gray arrows) and pseudo-cubic system (blue arrows) of NGO substrate. (**b**) The X-ray reciprocal space mapping (RSM) measurement around $[110]_{pc}$ for BFO(200Å)/LNO/NGO, where the reciprocal space units are normalized to 3.86Å.

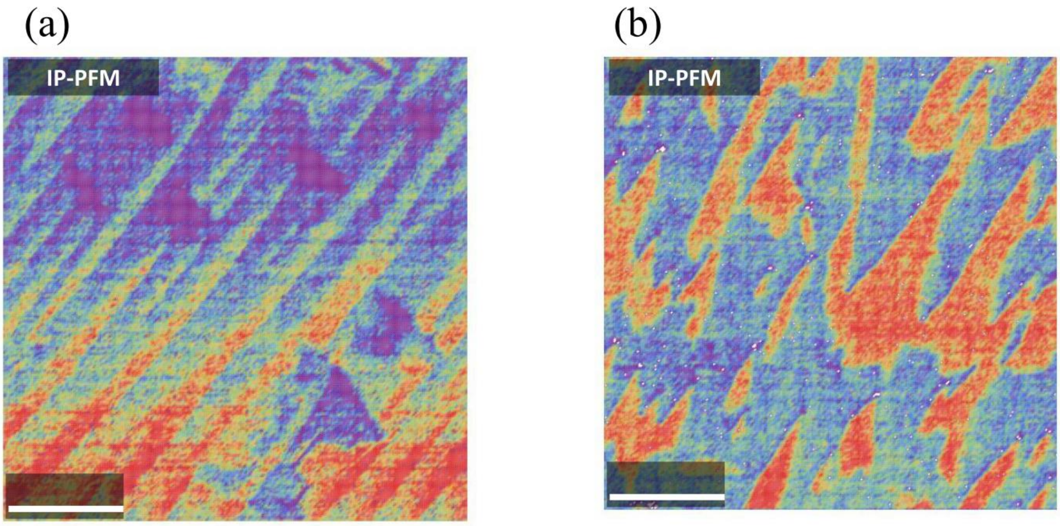

Supplementary Figure 10| Lateral piezoresponse force microscopy images (IP-PFM) of multi-domain BFO samples. **(a)** BFO(40Å)/$SrRuO_3$/$DyScO_3$ and **(b)** BFO(40Å)/$SrRuO_3$/$SrTiO_3$. Scale bars, 0.5um.

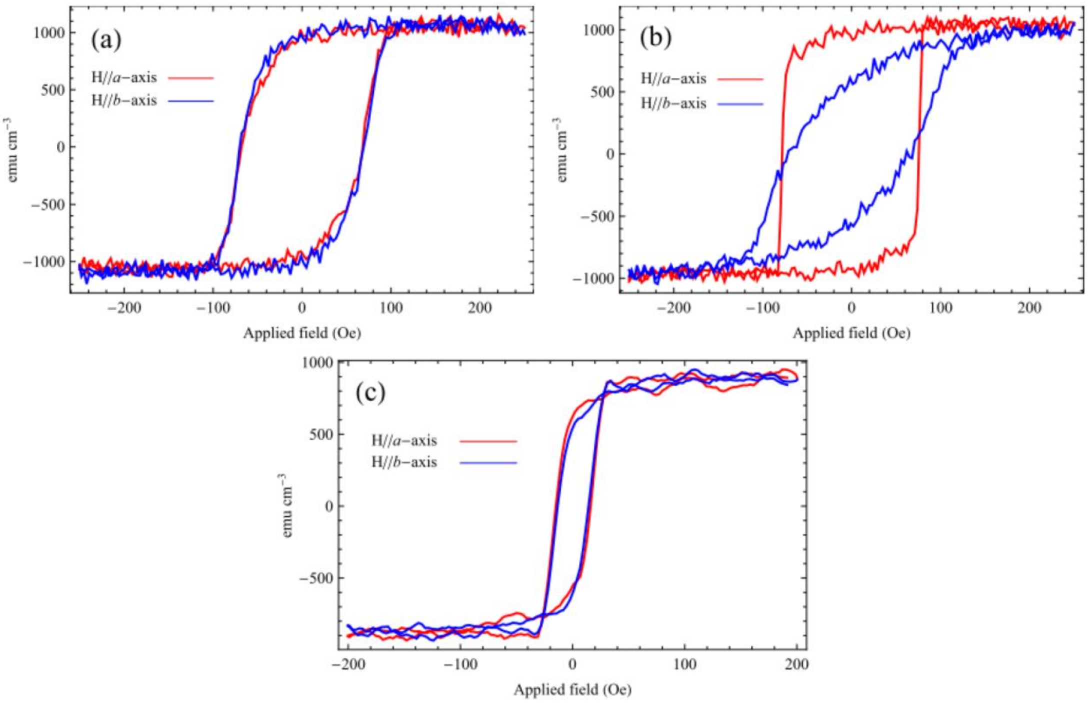

Supplementary Figure 11| Magnetic hysteresis curves curve of (**a**) Co(30Å)/ LNO/NGO, (**b**) Co(30Å)/BFO(40Å)/LNO/NGO, (**c**) Co(30Å)/BFO(200Å)/LNO /NGO.

Supplementary Note 1

AFM-axis orientation dependent calculations. Supplementary Figure 1 depicts the experimental Fe-L_2 XAS spectra of the BFO(40Å)/LNO/NGO film taken with **E**//**a** (black line) and **E**//**b** (red line). In the simulation we first put the spin direction (AFM-axis) along the b-axis, i.e. $\phi = 90°$ (see coordinate system in the inset). We can observe that the experimental spectra and the polarization dependence therein are well reproduced. Moving the spin direction away from the *b*-axis in the simulations, e.g. $\phi = 67.5°$, we obtain a set of calculated spectra with a less pronounced polarization dependence. For $\phi = 45°$ this polarization dependence vanishes completely. Moving the spin direction further towards $\phi = 22.5°$ and $\phi = 0°$, we obtain spectra which have a polarization dependence opposite to the experiment. These simulations therefore strongly suggest that the AFM-axis should be parallel or close to *b*-axis.

Supplementary Note 2

Angular dependence of XMLD. To determine the direction of the AFM-axis more precisely, we have measured the angular dependence of XMLD at Fe-L_2 XAS spectra. To quantify the magnitude of the polarization dependence, we measured the spectra for two orthogonal polarizations and define ΔLD as difference at peak A (LD_A) minus the difference at peak B (LD_B), see Supplementary Figure 1 for their definitions. First we carried out an experiment with the Poynting vector of the light (green arrow) perpendicular to the surface. We rotate the crystal around the *c*-axis, see Supplementary Figure 2(a), and plot the experimental ΔLD values (black dots) against ϕ', the (azimuthal) angle between the a-axis and one of the **E**-vector directions (yellow arrow). We then performed simulations with the spin direction (AFM-axis) parallel to the *b*-axis (red line) and parallel to the *a*-axis (blue line). We can directly conclude that the spin direction in the *ab*-plane is along the *b*-axis, and definitely not along the *a*-axis.

To check whether the AFM-axis is tilted away from the ab-plane or not, we have carried out also an experiment in which the Poynting vector (green vector) is at 20° grazing angle with the a-axis, see Supplementary Figure 2(b). We then rotated the crystal around this Poynting vector, and the angle between the **E** vector of the light (yellow arrow) and the *c'*-axis (the axis perpendicular to the Poynting vector and

close to the c-axis) is indicated by θ'. The measured ΔLD (black dots) as a function of θ' is plotted in Supplementary Figure 2(b), together with the simulations assuming the spin direction parallel to the *b*-axis (red line) and to the *a*-axis (blue line). Not only we confirm again that the experiment is well reproduced by the red line (and not by the blue line), but also that there is no out-of-plane component of the spin direction. Furthermore, we carried out an experiment where the Poynting vector of the light (green arrow) is at 20° grazing angle with the *b*-axis as shown in Supplementary Figure 3. We collected nearly identical spectra taken with the **E**//**a** and **E**//**c'** polarizations. This near-zero polarization dependence can be nicely reproduced by doing the calculations with the AFM-axis lying parallel to *b*-axis.

Supplementary Note 3

XMLD for AFM-axis along [101]. The XAS for **E**//**a** and **E**//**c'** are very similar. Such behavior could be interpreted in term of an AFM-axis along [101]. Supplementary Figure 4 shows the polarization dependent experimental and the calculated spectra with considering AFM-axis along [101]. One can see that the sign of calculated X-ray Magnetic Linear Dichroism (XMLD) is opposite to the experimental results with spin along [101]. Therefore, the AFM-axis cannot be along [101]. Furthermore, if the AFM-axis is along [101], one should expect a large XMLD signal between **E**//[101] and **E**//[-101]. We have measured the polarization dependent spectra for **E**//[101] and **E**//[-101] as shown in Supplementary Figure 5. One can clearly see that there is no XMLD signal at all. The results firmly confirm that AFM-axis does not lie along [101].

Supplementary Note 4

Piezo response force microscopy (PFM). In Supplementary Figure 7 we present the Band-Excitation PFM image for a 40Å BFO film grown on the NGO *without* bottom electrode. The BFO(40Å)/NGO shows the multidomain character. Vertical PFM contrast of BFO(40Å)/NGO can arise from cantilever buckling due to in-plane polarization components, and has been observed for different ferroelectric systems previously[1,2]. The presence of lateral PFM domains in addition to the vertical PFM in the same area suggests that the sample is both multi-domain, and contains in-plane polarization components. The bottom electrode thus plays another essential ingredient

to induce the single domain state in ultra thin BFO film.

Supplementary References

1. Nath, R. *et al.* Effects of cantilever buckling on vector piezoresponse force microscopy imaging of ferroelectric domains in $BiFeO_3$ nanostructures. *Appl. Phys. Lett.* **96,** 163101 (2010).

2. McGilly, L. J., Schilling, A. & Gregg, J. M. Domain bundle boundaries in single crystal $BaTiO_3$ lamellae: Searching for naturally forming dipole flux-closure/quadrupole chains. *Nano Lett.* **10,** 4200–4205 (2010).
